# Functional and Structural Characterization of SARS-Cov-2 Spike Protein: An *In Silico* Study

**DOI:** 10.4314/ejhs.v31i2.2

**Published:** 2021-03

**Authors:** Hadi Sedigh Ebrahim-Saraie, Behzad Dehghani, Ali Mojtahedi, Mohammad Shenagari, Meysam Hasannejad-Bibalan

**Affiliations:** 1 Razi Clinical Research Development Unit, Razi Hospital, Guilan University of Medical Sciences, Rasht, Iran; 2 Shiraz HIV/AIDS Research Center, Shiraz University of Medical Sciences, Shiraz, Iran; 3 Department of Microbiology, School of Medicine, Guilan University of Medical Sciences, Rasht, Iran

**Keywords:** SARS-CoV-2, Spike, Postmodification, Mutation, Bioinformatics

## Abstract

**Background:**

Severe acute respiratory syndrome coronavirus 2 (SARS-CoV-2) is the cause of the global outbreak of coronavirus disease 2019 (Covid-19), which has been considered as a pandemic by WHO. SARS-CoV-2 encodes four major structural proteins, among which spike protein has always been a main target for new vaccine studies. This in silico study aimed to investigate some physicochemical, functional, immunological, and structural features of spike protein using several bioinformatics tools.

**Method:**

We retrieved all SARS-CoV-2 spike protein sequences from different countries registered in NCBI GenBank. CLC Sequence Viewer was employed to translate and align the sequences, and several programs were utilized to predict B-cell epitopes. Modification sites such as phosphorylation, glycosylation, and disulfide bonds were defined. Secondary and tertiary structures of all sequences were further computed.

**Results:**

Some mutations were determined, where only one (D614G) had a high prevalence. The mutations did not impact the B-cell and physicochemical properties of the spike protein. Seven disulfide bonds were specified and also predicted in several N-link glycosylation and phosphorylation sites. The results also indicated that spike protein is a non-allergen.

**Conclusion:**

In summary, our findings provided a deep understanding of spike protein, which can be valuable for future studies on SARS-CoV-2 infections and design of new vaccines.

## Introduction

Coronaviridae is a family of enveloped, positive-sense singlestranded RNA viruses (ssRNA+) comprising coronaviruses for birds, bafiniviruses for fishes, and corona- and toroviruses for mammals (1). At the end of 2019, a series of pneumonia cases were reported from the Hubei Province of China with clinical presentations significantly resembling viral pneumonia (2). The etiology of infections was identified and confirmed as a novel coronavirus (2019-nCoV) belonging to β-coronavirus genera (2). The resulting virus and disease are currently called severe acute respiratory syndrome coronavirus 2 (SARS-CoV-2) and coronavirus disease 2019 (COVID-19), respectively (3).

There is limited information concerning the pathogenesis of COVID-19, and evidence has shown that the main mechanism is similar to SARS-CoV and MERS-CoV (4). The spike (S) protein of coronaviruses mediates viral entry into target cells. This entry is due to the binding of the surface unit (S1) of the S protein to a cellular receptor, known as angiotensin-converting enzyme 2 (ACE2). SARS-S and SARS-2-S share a high amino acid homology (>70%) (5). The interaction between viral proteins and cell membrane receptors is a critical step in the virus pathogenesis (6). The virus probably pass through major passages of the upper respiratory tract, especially nasal and larynx mucosa (7). The main target of virus entrance is lungs through the respiratory tract, but virus would also attack and enters other organs that express the type 2 transmembrane serine protease (TMPRSS2) and ACE2 receptor protein. The consequential of infection in host cells causes an excess release of pro-inflammatory cytokines that causes a cytokine storm (8).

COVID-19 patients exhibit various symptoms that cannot be easily distinguished from other respiratory diseases. Based on the severity of symptoms, this disease is classified into mild, moderate, severe, and critical (9). These symptoms, which may appear within a week after exposure to the virus, mainly include fever, cough, shortness of breath, chills, headache, muscle pain, and loss of taste or smell (10). The main reported complications associated with COVID-19 were pneumonia, heart injury, liver and kidney failure, and superinfections (11). Recent estimates showed that approximately half of died people with COVID-19 had a underlying diseases, where hypertension (46%) had the highest occurrence followed by diabetes (26%), cardiovascular disease (21%), malignancy (11%), chronic obstructive pulmonary disease (COPD) (8%), kidney disease (7%), and liver disease (3%)(12).

To date, there exists no specific antiviral treatment recommended for COVID-19, and no vaccine is currently available (13). The current appropriate treatments include oxygen therapy (which is the major intervention), administration of antibiotics to prevent bacterial co-infections, fluid management, and supportive use of traditional medicine (14,15). Other carried out strategies were using antivirals (Lopinavir, Ritonavir, Ribavirin, Favipiravir (T-705), Remdesivir, Oseltamivir, Chloroquine, and Interferon), and convalescent plasma (16). However, still the treatment effectiveness is greatly varied, so future studies on SARS-CoV-2 genome organization can help design and develop effective antiviral drugs or inhibition approaches.

Over the past decades, bioinformatics has emerged as a powerful tool for analyzing bacterial and viral genomes, predicting the structure and function of proteins, and designing new vaccines (17,18). Due to the global health emergency declared for COVID-19 and the importance of any effort to control the outbreak, the present *in silico* study aimed to investigate some physicochemical, functional, immunological, and structural features of spike protein using several bioinformatics tools.

## Materials and Methods

**Sequence alignment and phylogenetic tree**: All 52 SARS-CoV-2 spike protein sequences from different countries registered in NCBI GenBank (http://www.ncbi.nlm.nih.gov/) were retrieved from March to June 2020. The CLC Sequence Viewer Version Beta (Qiagen) was employed to analyze and detect the mutations in sequences. Phylogenetic tree was analyzed by UPGMA method (Bootstrap: 1000). The accession numbers of all sequences are displayed in Table 1.

**Table 1:** The accession numbers of all 52 sequences that were used in this study

Reference	NC_045512
China	MT281577, MT291829, MT291828, MT291826, MT291827, MT291830, MT291833, MT291832, MT291831, MT039874
USA	MT350236, MT350247, MT350269, MT350244, MT350237, MT350238, MT350252, MT350253, MT350254, MT350255
Iran	MT320891, MT281530
Japan	LC534418, LC534419, LC529905, LC528232, LC528233
India	MT050493, MT012098
Brazil	MT350282, MT126808
Nepal	MT072688
Italy	MT077125, MT066156
South Korea	MT304474, MT304475, MT304476, MT039890
Spain	MT292569, MT292570, MT292571, MT292572, MT292573, MT292574, MT292575, MT292576, MT292577, MT233523
Turkey	MT327745
South Africa	MT324062
Australia	MT007544

**Physicochemical properties**: Expasy's ProtParam (http://expasy.org/tools/protparam.html) was used to predict all the properties of spike protein, including theoretical isoelectric point (pI), extinction coefficient, instability index, molecular weight, aliphatic index, and grand average hydropathy (GRAVY)(19).

**Post-modification changes**: Serine, threonine, and tyrosine phosphorylation sites were predicted by DISPHOS (http://www.dabi.temple.edu/disphos/) and NetPhos (http://www.cbs.dtu.dk/services/NetPhos/), glycosylation sites were predicted by NetNGlyc (www.cbs.dtu.dk/services/NetNGlyc/) and Nglyde (http://bioapp.iis.sinica.edu.tw/Nglyde/run.php), and disulfide bonds were predicted using Dianna (http://clavius.bc.edu/∼clotelab/DiANNA/) and Scratch (http://scratch.proteomics.ics.uci.edu/) for spike protein.

**Immunoinformatics**: Chou and Fasman, Karplus and Schulz, Kolaskar and Tongaonkar, Emini, Parker, and BepiPred (http://www.iedb.org/) methods were applied to predict the position of B-cell epitopes. Hydrophilicity, flexibility/mobility, accessibility, polarity, exposed surfaces, and turns features were determined by BcePred (crdd.osdd.net/raghava/bcepred/). ABCpred (http://crdd.osdd.net/raghava/abcpred/) software specified 16 meric B-cell epitope. Allergenic properties were estimated using AlgPred (http://crdd.osdd.net/raghava/algpred/) and VaxiJen (http://www.ddgpharmfac.net/vaxijen/VaxiJen/VaxiJen) software which computed protective antigens and predicted subunit vaccines.

**Secondary and tertiary structures**: SOPMA software (https://npsaprabi.ibcp.fr/NPSA/npsa_sopma.html) and Phyre (http://www.sbg.bio.ic.ac.uk/∼phyre2/) server were applied to calculate and confirm the secondary structure, respectively. I-TASSER (https://zhanglab.ccmb.med.umich.edu/I-TASSER/) was utilized to predict the tertiary structure, and the suggested models were then refined by 3Drefine (http://sysbio.rnet.missouri.edu/3Drefine/).

Finally, the refined models were assessed for stereochemistry, reliability, and quality by “Qmean” (https://swissmodel.expasy.org/qmean/), “ProSA-web” (https://prosa.services.came.sbg.ac.at/prosa.ph), “ERRAT” (https://servicesn.mbi.ucla.edu/ERRAT/), and “Rammpage” (http://mordred.bioc.cam.ac.uk/∼rapper/rampage.php).

The study design was approved by regional Ethics Committee of Guilan University of Medical Sciences (IR.GUMS.REC.1399.001)

## Results

**Figure 1:**
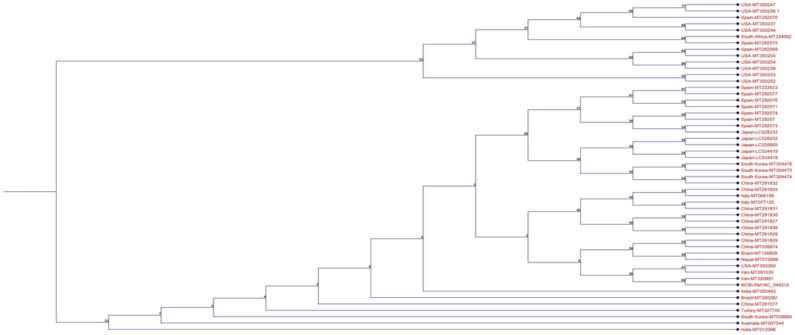
Phylogenetic tree of Spike protein sequences using neighbor joining method. The phylogenetic tree was constructed by the UPGMA method. The numbers at the forks show the numbers of occurrences of the repetitive groups to the right out of 1000 bootstrap samples.

**Amino acid changes:** Analysis showed that spike protein was a highly conserved protein, and only one high frequent mutation (D614G) was detected in comparison with the reference sequence. Table 2 summarizes all mutations established in spike protein. In addition, the phylogenetic tree results are illustrated in Figure 1. The phylogenetic analysis showed two main clusters, the upper one containing eight sequences form Spain, USA, and South Africa, and the second one including other sequences and reference sequences. Two sequences from Iran and a sequence form USA were very close to the reference sequence. Interestingly, almost all sequences from South East Asia (China, Japan, South Korea) were close to each other, and the majority of USA sequences were almost located in upper cluster.

**Table 2:** The list of substitutions were found in the present study and the high prevalent mutation is bolded

Mutations	Frequency No. (%)
S50L	1(1.9%)
N74K	1(1.9%)
S221w	1(1.9%)
S247R	1(1.9%)
R408I	1(1.9%)
**D614G**	**13(25%)**
V772I	1(1.9%)
A930V	1(1.9%)
V1065L	2(3.8%)

**ProtParam analysis**: ProtParam analysis indicated that spike protein is an acidic peptide due to the high percentage of its acidic amino acids (Theoretical pI: 6.2). The instability index, an estimate of the stability of a protein in a test tube, was 33.01 and showed that the spike was a stable peptide. Aliphatic index, a positive factor for the increased thermostability of proteins, this factor was 84.67 which revealed that this peptide was a thermostable one. GRAVY is a hydropathy index which augmented with the increase in the positive score. Thus, the peptide was also a hydrophilic one (-0.079).

**Postmodification and disulfide bond results**: Table 3 shows the postmodification and disulfide sites prediction; based on our results, the spike was highly phosphorylated, and four conserved positions were further suggested. Glycosylation prediction by two online software showed seven positions (61,74, 234, 282, 616, 709, and 1195); and results showed the prediction of possible disulfide bonds by Dianna and Scratch, which determined several cysteines.

**Table 3:** Postmodification and disulfide bond prediction results using several software

Software	NETPHOS	DISPHOS	Combined results
**Phosphorylation** **sites**	46, 50, 76, 95, 151, 170, 250, 255, 302, 313 359, 376, 415, 459, 469, 477, 523, 555, 572, 632, 637, 659, 680, 686, 730, 735, 813, 816 937, 939, 1003, 1105, 1147, 1155, 1196	250, 423, 572, 612, 680, 686, 811, 814, 817, 1048, 1262	250, 572, 680, 686

**Software**	**NetNGlyc 1.0** **Server**	**Nglyde**	**Combined** **results**
**Glycosylation** **Sites**	61,74,234,282,616,70 9,717,1159,1195	61,74,122,149,234,282,343,603, 616,657,709,1195	61,74,234,282, 616,709,1195

**Software**	**Dianna**	**Scratch**	**Combined** **results**

**Disulfide** **Bonds**	15 – 1241, 131 – 391, 136 – 662, 166 – 1237, 291 – 671, 301 – 336, 361 –488, 379 – 743, 432 – 1236, 480 – 1249, 525 – 1248, 538 – 1044, 590 – 617, 649 – 1242, 738 – 1244, 749 – 1127, 760 – 1255, 841 – 1033, 852 – 1251, 1083 - 1254	291–301, 1033–1044, 480–488, 379–391, 841–852, 1083– 1127, 15–1255, 525–538, 336–361, 662–671, 136–166, 131–1254, 590– 617, 738–749, 1236–1248	15, 131, 136, 166, 291, 301, 336, 361,379, 391, 480, 488, 525, 538,590, 617, 662, 671, 738, 749, 841, 852, 1033, 1044, 1083, 1127, 1254, 1255, 1236, 1248

**Figure 2:**
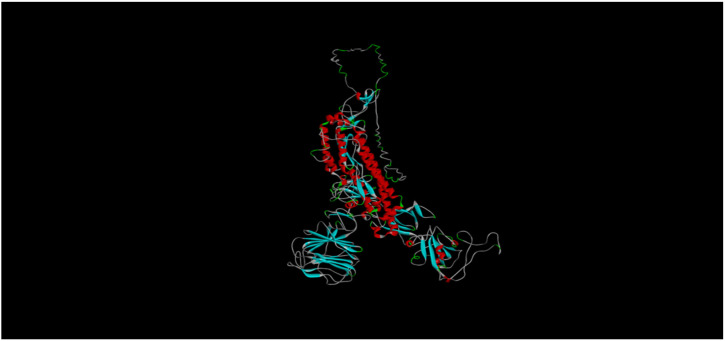
The final 3D structure of spike protein constructed by I-TASSER and refined by 4 online tools

**Secondary and tertiary structure prediction**: The secondary structure results using SOPMA showed that random coil was the major structure with 43.9% and after that Alpha helix, extended strand and Beta turn by 29.3%, 23.3% and 3.5% respectively. Table 4 presents the qualification results of the refined models suggested by 3D-refine. Figure 2 illustrates the tertiary structure of spike protein.

**Table 4:** The final results of 4 servers used to define the best tertiary structure for spike protein; selected structure in bold writing

Server	Models	Qmean	ERRAT	ProSA-web	Rampage (Ramachandran plot)	
I- TASSER 3D- Refine	without refinement	-10.01	71.371	not calculated	956 (75.2%)	188 (14.8%)
	1	-6.61	75.4772	-10.8	1010 (79.5%)	169 (13.3%)
	2	-6.03	74.7076	-11.07	1023 (80.5%)	154 (12.1%)
	3	-5.75	63.1799	-11.15	1039 (81.7%)	144 (11.3%)
	4	-5.75	60.6695	-11.17	1045 (82.2%)	138 (10.9%)
	5	**-5.62**	**61.5063**	**-11.27**	**1047 (82.4%)**	**135 (10.6%)**

**Immunoinformatics results**: The combination of all predictions provided by four software specified four B-cell epitopes (249–259,674–687,807–816, and 1254–1265). The VaxiJen score was 0.4646, indicating that this protein is a probable antigen. Algpred results suggested that spike protein is a non-allergen protein.

## Discussion

The results of the present study showed that the spike protein was highly conserved, and high prevalence mutation was detected only in one site (D614G). The mentioned mutation was observed amongst 90% of the sequences from USA, which was the highest rate among all countries. Analysis of other features of spike protein revealed that this mutation did not have any effect on post-modification sites, B-cell epitopes, and psychochemical properties. Banerjee et al. defined several mutations in the spike protein sequences from the USA, South America, China, and European countries (Banerjee et al. 2020) (20). Similar to our findings, substitution in amino acid 614 (D614G) was the most prevalent mutation (25%).

Previous studies suggested a region, KRSFIEDLLFNKV, as a potential Achilles' heel for controlling the life cycle of SARS-CoV-2. This site is exposed and this region is required for proteolytic activation cleavage (21,22). In addition, it is a well-conserved region located on the surface of the virus. Similar to previous investigations, our findings showed that KRSFIEDLLFNKV was completely conserved among all selected sequences from all regions. Interestingly, prediction of post-modification sites revealed that this region was phosphorylated. It was further predicted as a B-cell epitope, confirming its importance as a possible candidate for designing new vaccines.

Spike proteins contain a receptor binding domain (RBD) positioned between amino acids 331 and 524. Mutations in this region may critically impact virus entry and attachment to ACE2 receptor (23). In one sequence, we detected a substitution in this region, indicating that this domain is highly conserved and could be a new target for inhibiting virus attachment. Contrary to our predictions, Banerjee et al. specified four mutations (348, 476, 483, and 520) with very low prevalence (20). The difference between the two studies regarding the number of the mutations might be ascribed to the different sets of sequences and study methods.

Korber et al. focused on D614G substitution as an urgent concern, proposing that this mutation began spreading in Europe in early February 2020 (24). Although they were not able to define the origin of this mutation, there existed certain hypotheses as to its Chinese or European origin. The potential impacts suggested for this mutation are increased viral transmission, infected spike, enhanced receptor binding, and ADE (antibody-dependent enhancement) antibody elicitation(24). In agreement with Korber's study, our results indicated the spread of D614G substitution. Moreover, almost all sequences from North America (USA) and three sequences from Europe (Spain) harbored this mutation. Interestingly, this mutation was not detected in the sequences from China and South East Asia (Japan and South Korea).

Our analysis described spike protein as acidic, thermostable, and hydrophilic. However, because spike requires some post-modification processes, it seems yeast, and mammalian cells can better express this protein. Similar to our ProtParam prediction, Walls et al and Ou et al used different cell lines to express spike protein, which showed its stability in mammalian cells (25,26). Likewise, Zhang et al. expressed spike protein in *Escherichia coli*; they confirmed that *E. coli* was an appropriate host for the expression of spike (27).

Phosphorylation prediction showed four completely conserved sites among the selected sequences. Previous studies suggested some functions for protein phosphorylation in coronavirus. Petit et al proposed that phosphorylation is vital in the retention of spike protein at cell surfaces (28). Furthermore, Davidson et al stated that the phosphorylation sites on the spike glycoprotein might be necessary for assembling the trimer (29). Therefore, it can be concluded that blocking the phosphorylation process could be an effective approach to disturb the spike protein function.

Fung et al. defined the vital role of glycosylation in antigenicity, fusogenic, and immunomodulatory activities of the spike protein (30). Glycosylation prediction by NetNGlyc and Nglyde determined seven positions. Of these, except in the position from a Brazil sequence (74), which showed a substitution, other sites were highly conserved and seemingly highly vital to spike protein function. Shajahan et al .and Watanabeet al., using the high-resolution mass spectrometry, revealed 22 glycosylation sites for spike protein (31,32). Seven positions mentioned in our findings were similar to the foregoing studies. Similar to this study, Kumar et al. used bioinformatics tools to compare the glycosylation features of 2019-nCoV and SARS-CoV (33). In spite of different sequences used in the present study and Kumar's (Wuhan seafood market pneumonia virus isolate Wuhan-Hu-1 2019-nCoV-MN908947.3), the final predicted positions were completely similar. This shows that these positions remained unchanged during the spread of the novel coronavirus. By considering the critical role of glycosylation in the pathogenesis of SARS-CoV-2, these positions might be considered as new targets for novel inhibitors.

It has been proposed that disulfide bonds are required for a proper folding and trimerization of coronavirus spike protein (30). Dianna and Scratch results showed numerous positions for disulfide bonds that were completely conserved in all analyzed sequences. Dianna uses a support vector machine (SVM) with degree 2 polynomial kernel for the spectrum representation, and Scratch works based on 2D recurrent neural network, support vector machine, graph matching, and regression algorithms. Both online software are wellknown and were previously employed in numerous studies to define disulfide bonds. Ibrahim et al. made use of a combined molecular docking and structural bioinformatics; they detected 13 disulfide bonds in four distinct regions and suggested that these regions were involved in cell attachment (34). Despite the different sequences and methods used in Ibrahim's study and the present one, all predicted bonds were similar.

Previous studies confirmed the humoral immune response against spike protein in infected patients (Tay et al. 2020; Temperton et al. 2005; Yuchun et al. 2004) (35–37). It was shown that neutralizing antibody responses to the spike protein began by week two and in most patients developed by week three. Immunoinformatics analysis of spike protein by certain online databases suggested four regions that confirmed the possible potential of this protein for inducing humeral immune system. Interestingly, no mutation was detected in these regions; hence, they could be proper regions for the production of new vaccines. In addition, estimating allergenic characteristics showed that spike protein could not provoke allergenic reactions in humans.

Ahmed et al. used bioinformatics approaches to define B-cell epitopes in different proteins of SARS-CoV-2 (38). They were able to define 23 B-cell epitopes for spike protein; our prediction, on the other hand, showed new regions, a difference possibly attributable to the different sequences used in both studies.

Moreover, through bioinformatics analysis and machine learning, Grifoni et al. and Fast et al. analyzed spike protein to define immunological properties. Compared with our prediction (39,40), the two sites (674–687 and 807–816) were similar.

As a major limitation of this study, information on the COVID-19 crisis is constantly changing, and day-by-day number of new sequences in online databases are updated, therefore our study may not present a comprehensive view of spike protein. However, as a preliminary study, our results provide an insight for further works.

In summary, the results of the present study provided a comprehensive understanding of spike protein which can be used for further studies. This protein is a highly capable epitope on the SARS-CoV-2 surface which included several features appropriate in a vaccine construct. Other features of spike protein could be employed to express this protein, and post-modification sites could be utilized as new targets for SARS-CoV-2 inhibitors. Meanwhile, it is not easy to forecast any realistic scenario, but mutations in spike protein suggest potential impacts on the pathogenesis of the virus in near future.
